# Evaluation of a training program for medicines-oriented policymakers to use a database of systematic reviews

**DOI:** 10.1186/s12961-016-0140-1

**Published:** 2016-09-21

**Authors:** Heather L. Colquhoun, Dianne Lowe, Eftyhia Helis, Denis Belanger, Brendalynn Ens, Sophie Hill, Alain Mayhew, Michael Taylor, Jeremy M. Grimshaw

**Affiliations:** 1 Department of Occupational Science and Occupational Therapy, University of Toronto, 160-500 University Ave, Toronto, ON M5G 1V7 Canada; 2 Centre for Health Communication and Participation, School of Psychology and Public Health, College of Science, Health and Engineering, La Trobe University, Melbourne, 3086 Australia; 3 Knowledge Mobilization and Liaison Officer Program, Canadian Agency for Drugs and Technologies in Health (CADTH), Ottawa, ON Canada; 4 C.T. Lamont Primary Health Care Research Centre, Bruyère Research Institute, 43 Bruyère Street, Annex E – 208, Ottawa, ON K1N 5C8 Canada; 5 Public Health, School of Allied Health, Australian Catholic University, Fitzroy, VIC 3065 Australia; 6 Department of Medicine, University of Ottawa, Epidemiology & Community Medicine, 451 Smyth Road, Ottawa, ON K1H 8M5 Canada; 7 Clinical Epidemiology Program, Ottawa Hospital Research Institute, Ottawa, ON Canada

**Keywords:** Evidence-informed, Systematic reviews, Integrated knowledge translation, Prescribing medicines, Rx for Change

## Abstract

**Background:**

Suboptimal prescribing and medications use is a problem for health systems globally. Systematic reviews are a comprehensive resource that can help guide evidence-informed decision-making and implementation of interventions addressing such issues; however, a barrier to the use of systematic reviews is their inaccessibility (due to both dispersion across journals and inaccessibility of content). Publicly available databases, such as *Rx for Change*, provide quick access to summaries of appraised systematic reviews of professional and consumer-oriented interventions to improve prescribing behaviour and appropriate medication use, and may help maximise the use of evidence to inform decisions. The present study aims to evaluate a training program to improve attitudes towards, confidence in skills, intentions to use, and use of systematic review evidence contained within *Rx for Change*.

**Methods:**

Guided by the *Knowledge to Action* framework, a training program with content customised to local provider and consumer contexts was developed with knowledge user input. The training program consisted of a 6 minute information video, a 1 hour workshop with hands-on, interactive and didactic components, and two post-training reminders. Forty-nine people from five medicines-focused organisations in Canada and Australia attended one of six workshops. Participants were surveyed immediately pre and post and 3 months after training to evaluate their attitudes towards, confidence in skills, intentions to use, and use of *Rx for Change*, and attitudes towards and confidence in skills for using evidence for decision-making. Analyses for differences for each of the outcomes at three time points (pre, post and 3 months after training) was performed using a random effects model.

**Results:**

Immediately post-training, there were higher respondent attitudes towards *Rx for Change* (mean increase = 0.54 out of 5, 95% CI, 0.18–0.83, *P* < 0.005); intention to use *Rx for Change* (0.53, 95% CI, 0.21–0.86, *P* < 0.005); confidence in skills for using *Rx for Change* (2.08, 95% CI, 1.74–2.42, *P* < 0.005); and confidence in skills for using evidence in policy decision-making (0.50, 95% CI, 0.22–0.77, *P* < .005) compared to pre-training. Confidence in skills for using both *Rx for Change* and evidence were maintained 3 months after training (both *P* < 0.005).

**Conclusions:**

Participants of this training program reported sustained improvements in their confidence in skills for using evidence in policy decision-making. This may have important implications for uptake of systematic review evidence promoting improved prescribing and medication use.

## Background

Health systems globally are focused on addressing inappropriate prescribing by healthcare professionals (providers) and inappropriate use of medications by patients (consumers) to maximise patient outcomes and minimise healthcare costs [1, 2]. Sub-optimal medication use and prescribing practices exist across most high-income countries [1, 3], with medication errors, mistakes and laboratory error in Australia and Canada being reported for approximately 27% and 30% of patients, respectively [1]. Poor prescribing practices and medication errors are associated with negative health outcomes, including increased morbidity [4] and preventable hospital admissions [5], increased rates of antibiotic resistant organisms due to overprescribing of antibiotics [6] and high rates of preventable adverse drug events [7]. Suboptimal medication use by patients is also problematic, with adherence to medicines rates of approximately 50% [2]. Problems affecting consumers’ use of medicines include high rates of errors by prescribers and patients, preventable adverse effects, and difficulties in communication and transitional care [8].

Systematic reviews comprehensively synthesise available evidence about potential policies and interventions to improve medication use but knowledge users find it difficult to access relevant systematic reviews and make sense of research evidence [9]. Knowledge users also face significant challenges to using systematic review evidence. Some of the most frequently identified barriers by knowledge users include constraints on time and resources, and resistance towards change [10]. Knowledge users report that increasing the use of research evidence for policy decision-making requires more accessible and efficient systems as well as approaches to ensuring accountability for evidence use [10]. There are also barriers related to the context in which policy decision-making occurs, including varying pressure from multiple interest groups, limited time for decision-making and the use of research evidence being one of many types of information required for policy decision-making [9]. Further barriers to evidence-informed decision-making include lack of senior-level leadership and support for using evidence, politicised (as opposed to evidence) decision-making and constant shifting of attention across changing priorities making a focus on evidence impossible [11].

In response to these challenges, a range of innovative knowledge translation resources have been developed to facilitate the use of evidence to inform decision-making, including simplified frameworks developed with contribution from the policy sector to better understand how any given approach to increasing the use of evidence can be applied and understood [12], websites to facilitate research engagement by policymakers [13], and databases to facilitate access to evidence [14, 15]. The Cochrane Collaboration has developed training courses for systematic review use specific to public health [16] and overviews of systematic reviews have been published to facilitate summaries of evidence [8, 17].

One such freely available online database is *Rx for Change*, an evidence-based resource of appraised and summarised evidence on the effectiveness of interventions targeting healthcare professionals and patients to improve prescribing and medication use [14] (http://www.rxforchange.ca). *Rx for Change* is a collaboration between researchers at the Cochrane Effective Practice and Organisation of Care (EPOC) Group and the Cochrane Consumers and Communication Review Group (CC&CRG) primarily funded by the Canadian Agency for Drugs and Technologies in Health [14]. Between 2007 and April 2014, the database was periodically updated to ensure that this evidence remains relevant and timely. To populate the database, potentially relevant systematic reviews of interventions targeting professionals (e.g. audit and feedback) and consumers (e.g. acquiring skills and competencies) were identified by sensitive searches of major databases including Medline, *The Cochrane Library*, and EMBASE. Full text copies of potentially relevant reviews were retrieved and assessed for relevance [8]. Each review was quality-assessed using AMSTAR, an instrument validated for assessing quality of systematic reviews [18–20]. High and medium quality reviews (AMSTAR score > 4) were data-extracted and summarised using a structured process to highlight key characteristics and evidence relevant to optimal use of medicines decision-making. To facilitate the ease of finding and using information, the *Rx for Change* database is organised according to intervention type (e.g. education, reminders, etc.) and category (e.g. professional, consumer, organisational, financial, regulatory) using taxonomies developed by EPOC and CC&CRG [21]. Currently, *Rx for Change* contains more than 300 summarised reviews that synthesise findings from thousands of individual studies, spanning all conditions and diseases, and evaluating interventions targeting healthcare provider or consumer behaviours to improve medication prescribing or use.

The primary objective of *Rx for Change* is to maximise the awareness and use of appraised systematic reviews of professional and consumer-oriented interventions to improve prescribing behaviour and appropriate medication use. There has been a steady trend of increased site visits and time spent on the site since its inception and the database has received positive feedback from healthcare providers, researchers and policymakers [14]. However, despite efforts to increase the use of *Rx for Change* (e.g. an on-line tutorial), anecdotal evidence suggests the need for further training to maximise use of the database for evidence-informed decisions.

### Objectives

The aim of this project was to develop and evaluate a training program for *Rx for Change* in collaboration with relevant knowledge users. The development of the training was guided by the Knowledge to Action framework [22]. This framework outlines the necessary steps to bridge the gap between knowledge and the application of that knowledge in healthcare settings. The framework steps emphasise collaboration with knowledge users and includes adapting knowledge to the local context, understanding local barriers and tailoring interventions as needed. The comprehensive findings of the development stage of the training intervention (Phase 1), which involved interviewing 16 knowledge users from a variety of settings to identify the needs of the organisations and to guide the content, delivery and tailoring of the workshop, are presented elsewhere [23]. The focus of the current study (Phase 2) was to evaluate the resultant training program in terms of its impact on subsequent attitudes towards, confidence in skills, intentions to use, and use of *Rx for Change*, as well as attitudes towards and confidence in skills for using evidence in general.

## Methods

This study used a pre–post design with a 3-month follow-up. Five knowledge user organisations responsible for the development of programs to improve prescribing and medication use contributed to this project, three from Canada (provider focused) and two from Australia (consumer focused). All five of these organisations expressed an interest in improving their use of *Rx for Change* and a need for additional training. The three Canadian sites focused on evidence for organisational, professional and regulatory interventions, which are consistent with the primary interest (and expertise) of EPOC. Consistent with the primary interests (and expertise) of CC&CRG, the knowledge user organisations from the two Australian sites focus primarily on evidence for consumer-oriented interventions. The organisations were a mix of national (n = 2), provincial (n = 3), and government (n = 4) or non-government (n = 1) sites. All programs provided support and information that was needs driven, with the Canadian sites focusing on the ultimate goal to influence local provider behaviour, and the Australian sites focusing on consumer behaviour.

### Stage 1

#### Training development

Key informants (n = 16) from each knowledge user organisation participated in interviews to shape the content and format for the training programs so that they were (1) tailored to their needs and (2) designed to address provider or consumer issues and barriers across the two countries. The semi-structured interviews included questions on their current use of evidence for decision-making, barriers and facilitators to using evidence as well as the *Rx for Change* database, and their needs and preferences for training. The interviews were transcribed and analysed using a directed content analysis [24]. The main themes from the interviews identified the need for the workshops to cover specific knowledge (e.g. definition of a systematic review), skills for both navigating the database as well as how to incorporate evidence into decision-making, activities to promote the routine use of *Rx for Change*, and the need to address a variety of preferences for how the training should be delivered (tailoring). There was also an interest in including practical and locally important examples on incorporating evidence within their workplace context. In order to respond to this need and the desire to learn how to use evidence for policy decision-making, we developed a workshop that incorporated content from an existing framework for using evidence in policy decision-making – the SUPPORT Tool process [25]. The SUPPORT Tool process includes the following steps: identifying the problem and/or question at hand, What do I want to achieve?; identifying the available options to address the problem; identifying the available resources; decision-making; and implementation of most suitable options. The developed training program consisted of (1) a 6 minute information video to be viewed prior to the workshop (the video provided basic background information about the database and systematic reviews); 2) a 60 minute face-to-face workshop with didactic, hands-on and interactive components (these components included a 30 minute skills demonstration and practice with the database that took participants through the SUPPORT Tool process using a local and current prescribing-based issue in the organisation); and (3) post workshop reminders at 1 and 3 months following the workshop to remind participants about *Rx for Change* and encourage its use. Following the development of the training, a final feasibility check for the proposed training was completed with a manager from each of the five participating organisations. A more comprehensive presentation of the findings of the interviews and details of intervention design that resulted from this initial stage of the project is presented elsewhere [23].

### Stage 2

#### Training program implementation and evaluation

Participants were recruited from each of the knowledge user organisations via email invitations distributed to one key contact within each of the organisations and forwarded to potentially interested staff. The training was delivered to the five knowledge user organisations (training was provided twice to one organisation with a significant geographic range). Participants were surveyed immediately pre and post and 3 months after training. The surveys included questions on self-reported use of *Rx for Change* in the 3 months prior to the workshop and in the 3 months following the workshop (i.e. how many times in the past three months had they used *Rx for Change*), attitudes towards using evidence and *Rx for Change* for policy decision-making (i.e. their rating on a scale from 1–5 as to the appropriateness, importance and usefulness of both evidence and *Rx for Change*), confidence in skills (i.e. rating on a scale of 1–5 for level of confidence in six relevant skills for using evidence and 11 relevant skills for using *Rx for Change*), and intention to use *Rx for Change* (i.e. global rating of intention on a scale from 1–5). Surveys were developed for the study and were piloted in terms of relevancy, clarity and completeness.

Given the large number of variables in the survey (i.e. 24), the problems associated with multiple testing [26], and the fact that our survey contained many similarly phrased questions for attitudes and skills, we planned a principal component analysis of groupings of questions in order to determine if the data supported the use of a mean score for each of these groupings. The resulting principal component analysis indicated that the groupings of questions were highly correlated – all corrected item-total correlations were above the recommended threshold of r > 0.30 [27]. We therefore, used these groupings, and the associated mean scores, for the analysis under the four labels of (1) attitudes towards *Rx for Change*, (2) confidence in skills for using *Rx for Change*, (3) attitudes towards using evidence in decision-making, and (4) confidence in skills for using evidence for decision-making. Table 1 provides a detailed summary of this analysis. Combined with intention (analysed as a single variable), this gave us five outcomes to test.

**Table 1:** Results of the principal component analysis

Grouping label	Questions (5-point Likert scale)	Principal component analysis (r value)
Attitudes about evidence for decision-making	Answer the following: 1. I think that using evidence in my decision-making is appropriate 2. I think that using evidence in my decision-making is important 3. I think that using evidence in my decision-making is useful	All > 0.88
Confidence in skills at using evidence for decision-making	Rate level of confidence in the following skills: 1. Formulate an answerable question about an issue or topic related to my work, to guide an online evidence search 2. Conduct an online evidence search to address the question 3. Critically appraise the research evidence 4. Determine how the evidence should be applied and transferred to my own context 5. Overcome barriers in using evidence in my workplace 6. Overall confidence in using evidence in decision-making	All > 0.66
Attitudes about *Rx for Change* for decision-making	Answer the following: 1. I think that using *Rx for Change* in my decision-making is appropriate 2. I think that using *Rx for Change* in my decision-making is important 3. I think that using *Rx for Change* in my decision-making is useful	All > 0.97
Confidence in skills at using *Rx for Change* for decision-making	Rate level of confidence in the following skills: 1. Finding the *Rx for Change* database online 2. Navigating within the database 3. Knowing how best to use the “search” function 4. Finding the intervention level summaries 5. Understanding the intervention level summaries 6. Finding the review level summaries 7. Understanding the review level summaries 8. Knowing where to find reviews 9. Knowing where to find the individual studies in the reviews 10. Understanding the review quality score 11. Interpreting the *Rx for Change* evidence so that I can apply it in my own context	All > 0.62

### Data analyses

The survey responses were analysed descriptively and with frequencies and counts. Differences before and after training as well as between provider and consumer oriented organisations were analysed using a random effects model, with organisational unit used as a random effect and robust variance estimates used for each of the five outcome measures, each assessed on a scale from 1 to 5 time period (pre, post and 3 month follow-up) was the exposure variable, with the pre-training time point being used as the reference point. As participant responses were anonymised at the point of collection, repeated measures analyses could not be performed. To formally test if the effect of the intervention may vary between countries or organisations, interaction terms were fitted between time points and these clusters. A Bonferroni correction for multiple analyses (10 comparisons, two each for five outcome variables) was used, resulting in a *P* value of < 0.005 being considered as significant. All statistical analyses were undertaken using Stata (v13.1, College Station, Tx).

## Results

**Fig. 1:**
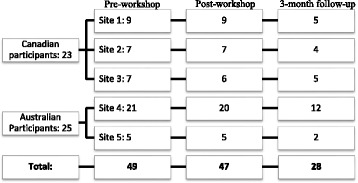
Number of participant responses for pre, post and follow-up surveys at each site

A total of 49 participants attended the workshops and submitted the pre-workshop survey (one with incomplete responses). For the post-training survey and 3-month follow-up, we had 47 and 28 participants submit the survey, respectively (two and one with incomplete responses, respectively). Figure 1 provides a detailed summary of countries, organisations and surveys completed for all time points. The participant sample was comprised of 79% females (38 female, 10 male) and 88% full-time workers (42 full-time, 6 part-time). Of the participants attending the workshops, 60% (29/48) had previously heard about the *Rx for Change* database.

### Self-reported use of *Rx for Change*

Prior to the training, 78% (38/49) of participants indicated that they never used *Rx for Change* in the preceding 3 months. When asked to consider their use of *Rx for Change* in the 3 months since the training, only 39% (11/28) indicated that they never used *Rx for Change*. The number of people indicating that they used *Rx for Change* in the previous 3 months at least 2–3 times per month increased from 8% (4/49) during the 3 months prior to the training to 32% (9/28) during the 3 months after the training. Table 2 shows the full summary of self-reported use of *Rx for Change*.

**Table 2:** Self-reported use of *Rx for Change* in 3 months pre and post the workshop

Answer to the question ‘how many times have I used *Rx for Change*…	….in the 3 months prior to workshop’ Count (%)	….in the 3 months since the workshop’ Count (%)
	n = 49	n = 28
Never or not applicable	38 (78%)	11 (39%)
Once	7 (14%)	8 (29%)
Twice	4 (8%)	5 (18%)
Three times (monthly)	0 (0%)	4 (14%)
Twelve times (weekly)	0 (0%)	0 (0%)

### Statistical analysis for group differences

#### Differences in pre to post and pre to 3 month follow-up

Results indicated that confidence in skills to use *Rx for Change* and confidence in skills to use evidence were statistically significantly greater than pre training at both the post and 3 month follow-up time points (all *P* < 0.005). Attitudes towards using evidence for decision-making were not significantly different from pre training at post training (*P* = 0.635) or at 3 months follow-up (*P* = 0.662). Both attitudes towards, and intentions to use, *Rx for Change* increased at post training (both *P* < 0.005), but not at follow-up (*P* = 0.445 and *P* = 0.908, respectively). Table 3 provides a summary of the analysis for group differences. There was no evidence that either the organisational unit or country modified these associations (all *P* values for interactions > 0.01), although there was limited power to detect such effects.

**Table 3:** Statistical Analysis for Group Differences

	Pre – time point mean (95% CI)	Post – time point mean difference from pre (95% CI) *P* value	3 month – time point mean difference from pre (95% CI) *P* value
	n	n	n
Attitudes towards using evidence for decision-making^a^	4.75 (4.59 to 4.91) n = 49	−0.05 (−0.27 to 0.17) *P* = 0.635 n = 46	0.06 (−0.21 to 0.33) *P* = 0.662 n = 28
Confidence in skills for using evidence^b^	3.60 (3.40 to 3.81) n = 49	0.50 (0.22 to 0.77) *P* < 0.005 n = 45	0.53 (0.23 to 0.82) *P* < 0.005 n = 28
Attitudes towards using *Rx for Change* in decision-making^c^	3.95 (3.67 to 4.22) n = 49	0.50 (0.18 to 0.83) *P* < 0.005 n = 46	−0.17 (−0.61 to 0.27) *P* = 0.445 n = 28
Confidence in skills for using *Rx for Change* ^d^	2.38 (2.09 to 2.67) n = 48	2.08 (1.74 to 2.42) *P* < 0.005 n = 47	1.74 (1.37 to 2.11) *P* < 0.005 n = 28
Intention to use *Rx for Change*	3.87 (3.52 to 4.21 n = 48)	0.53 (0.21 to 0.86) *P* < 0.005 n = 46	−0.06 (−0.51 to 0.45) *P* = 0.908 n = 27

^a^Mean of three questions on perceptions of evidence use as appropriate, important and useful

^b^Mean level of confidence for six skills in using evidence for decision-making

^c^Mean of three questions on perceptions of *Rx for Change* as appropriate, important and useful

^d^Mean level of confidence for 11 skills in using *Rx for Change*

## Discussion

We developed user-informed training to increase the use of an online database of systematic reviews (*Rx for Change*) that policymakers could use to inform the design of programs to improve medication use. We tested this training for effects on attitudes towards, confidence in skills, intentions to use, and use of *Rx for Change* as well as attitudes towards and confidence in skills for using evidence for decision-making. We measured our outcomes pre and post the training workshop as well as at 3 months post training. Pre–post training effects were found for attitudes towards, and confidence in skills for using *Rx for Change*, as well as confidence in skills for using evidence for decision-making. More importantly, effects on confidence in skills for using *Rx for Change* and for using evidence for decision-making were maintained at 3 months post training. Therefore, the results indicate that this workshop, developed with input from knowledge users and using an evidence-based process of using evidence for policy decision-making as a guide, has potential to improve immediate and long-term confidence in skills in using this particular database, and in using research evidence in general.

We combined several evidence-based approaches in the design of our training. We used a workshop that incorporated interactive components [28], content tailored to the needs and preferences of the knowledge users [29], reminders [30], and the integration of knowledge user input [22]. Systematic review evidence indicates that each of these approaches has been shown to have an effect on behaviour, albeit modest. Our training appears to have resulted in increased confidence in skills and increased use of *Rx for Change*, although it did not have an effect on intention, thus reducing the likelihood of sustaining the increased use of *Rx for Change* [31]. The use of a 1-hour workshop is a common approach to education and behaviour change [32] and the leads in our knowledge user organisations had a preference for this style of training as it fits with demands placed on staff in these organisations. While more understanding is needed as to dosage and the types of educational activities for maximising effects, it continues to be worthwhile to test the effects of short educational training interventions and ensure follow-up measures to optimise our understanding of the maintenance of any gains made.

There were no significant differences observed in attitudes towards using evidence for decision-making at any time point. Given the high pre-training means for this variable (4.74 on a 5 point scale), there was either little room for improvement (suggesting a ceiling effect) or our scale was not able to detect differences. Attitudes towards using evidence tend to be positive, even if behaviour is not consistent with this attitude. Programs of this nature should probably not solely focus their efforts on changing attitudes towards using evidence but rather behaviours around the use of evidence. We did not observe any differences in intentions to use *Rx for Change* at the 3 month follow-up. The reasons for this are unclear, but could include perceptions of the value and/or functionality of the database itself or broader issues related to the use of evidence in general. Although we did incorporate the needs and preferences of the knowledge users into the training, we focused on this local input, perhaps at the expense of considering other barriers to change such as organisational culture [33]. While a 1 hour workshop can only cover a limited amount of content, future efforts to address knowledge user needs as well as other barriers identified in the literature is warranted.

Forty percent of the sample indicated that they had not used *Rx for Change* since the training, yet there were fewer participants who indicated that they did not use *Rx for Change* in the 3 months after training than in the 3 months prior to training. We were unable to explore if participants had a need to use *Rx for Change* since the training, making it difficult to fully interpret our *Rx for Change* use data. However, the results suggest that with no change in their intentions to use *Rx for Change*, a decrease in their attitudes towards it, and confidence in skills potentially declining, it is likely that use rates will revert to pre-training levels. This is often the case in knowledge translation interventions [34], and methods for promoting knowledge translation intervention sustainability are needed.

Confidence in skills for using evidence improved after the workshop, with the improvement being maintained 3 months later. This is an important result, particularly for a 1 hour workshop. Similar to confidence in skills for using evidence, the workshop improved perceptions of confidence in skills for using *Rx for Change*, and this was maintained at 3 months. However, there is a trend towards declining confidence in skills. This might signal that additional skill development activities might be required to support the ongoing use of a database like *Rx for Change*.

While consumer-based and provider-based organisations from different countries were used in the study, the staff within these organisations undertook similar duties – outreach to others to improve use of evidence. The ‘others’ were different but the people we targeted with the intervention were not that different. If, for example, physicians had been the targets of the education, differences would have been expected. This finding might improve the generalisability of our findings to a broad array of policy-based groups. However, caution in generalising is warranted given our context of only two countries and both being of high income.

We guided our research using the Knowledge to Action framework [22], which gave us a structure that underpinned the development, pilot and evaluation of this multi-faceted training program to improve use of *Rx for Change* [23]. Another strength of this study is that the research builds on international collaborations between Canada and Australia by involving five knowledge user groups who have expressed the interest in *Rx for Change* training, three from Canada and two from Australia. By involving targeted user audiences we can better understand the needs of individuals or groups concerned with the identification and/or implementation of interventions used to change provider (health practitioner/professional) and consumer (patient) behaviours as it specifically relates to medicines prescribing or practice. Limitations of this study include a relatively small number of sites and participants. In addition, the 3 month follow-up data only included 28 of the 49 participants who attended the workshop, resulting in an even smaller sample at the follow-up, thus increasing the potential for bias and reducing power to observe effects. While we did include a 3 month follow-up, the timing of this follow-up was short, and no further follow-up took place. The most significant limitations were the design of the study (a pre–post measurement with no control group and no ability to measure changes in responses over time within individuals) and the use of a survey that was designed specifically for the study. While we did pilot test the survey for relevancy, clarity and completeness, we did not undertake additional measures of reliability and validity. Our conclusions are therefore tentative and additional experimental research is needed.

## Conclusion

The evaluation component aids understanding and future activities to optimise use of *Rx for Change*. Participants of this tailored training program reported sustained improvements in confidence in skills for using evidence in policy decision-making. This may have important implications for uptake of systematic review evidence to promote improved prescribing and medication use.

## Abbreviations

CC&CRG: Cochrane Consumers and Communication Review Group
EPOC: Effective Practice and Organisation of Care
